# Antidiabetic and antioxidant properties of *Ficus deltoidea* fruit extracts and fractions

**DOI:** 10.1186/1472-6882-13-118

**Published:** 2013-05-29

**Authors:** Hasni Misbah, Azlina Abdul Aziz, Norhaniza Aminudin

**Affiliations:** 1 Department of Molecular Medicine, Faculty of Medicine, University of Malaya, 50603, Kuala Lumpur, Malaysia; 2 University Malaya Centre for Proteomics Research (UMCPR), Medical Biotechnology Laboratory, Faculty of Medicine, University of Malaya, 50603, Kuala Lumpur, Malaysia; 3 Institute of Biological Sciences, Faculty of Science, University of Malaya, 50603, Kuala Lumpur, Malaysia

**Keywords:** Antioxidant, Diabetes, *Ficus deltoidea*, Phenolic, SDS-PAGE, SELDI-TOF MS

## Abstract

**Background:**

Diabetes is a serious metabolic disorder affecting the metabolism of carbohydrate, protein and fat. A number of studies have shown that diabetes mellitus is associated with oxidative stress, leading to an increased production of reactive oxygen species. *Ficus deltoidea* is traditionally used in Malaysia for regulating blood sugar, blood pressure and cholesterol levels. The use of *F*. *deltoidea* as an alternative medicinal herb is increasingly gaining popularity with the sale of *F*. *deltoidea* tea bags and capsules in the local market. The present study was undertaken to investigate the antidiabetic and antioxidant activities of the fruits from different varieties of *F*. *deltoidea*, employing *in vitro* methods.

**Method:**

Two fruit varieties of *F*. *deltoidea* (var. *angustifolia* (SF) and var. *kunstleri* (BF)) were extracted separately using double-distilled water. The resulting aqueous extracts were partitioned using ethyl acetate to obtain the ethyl acetate and water fractions. The crude aqueous extracts and the corresponding fractions were evaluated for their phenolic, flavonoid, sugar and protein contents. Protein profiling of the extracts and fractions were also carried out by means of SDS-PAGE and SELDI-TOF MS. Antidiabetic activities were assessed based on the ability of the samples to inhibit yeast and mammalian α-glucosidase as well as α-amylase. Antioxidant capacities were examined by measuring the ability of the samples to reduce ferric ions and to scavenge DPPH, superoxide anion, ABTS and nitric oxide radicals.

**Results:**

The crude extracts and fractions of SF and BF inhibited both yeast and rat intestinal α-glucosidases in a dose-dependent manner, but did not inhibit porcine pancreatic α-amylase. The water fraction of BF showed the highest percentage of α-glucosidase inhibition while having the highest amount of protein (73.33 ± 4.99 μg/mg fraction). All the extracts and fractions exhibited antioxidant activities, with SF crude extract showing the highest antioxidant activity and phenolic content (121.62 ± 4.86 mg/g extract). Fractionation of the crude extracts resulted in loss of antioxidant activities. There was no positive correlation between phenolic and flavonoid content with α-glucosidase inhibitory activities. However, phenolic content correlated well with antioxidant activities of the crude extracts but not with the fractions.

**Conclusions:**

The antioxidant activities of the fruits of *F*. *deltoidea* might be asserted by the phenolic content but other polar plant components were possibly involved in the antidiabetic properties. The study of these compounds having both antihyperglycemic and antioxidant activities may provide a new approach in the treatment of diabetes mellitus.

## Background

Diabetes mellitus is a metabolic disease characterized by hyperglycemia and disturbances in fat and protein metabolism that results from defects in both insulin secretion and/or insulin action [1]. Various pharmacological approaches have been used to improve diabetes via different modes of action including stimulation of insulin release, inhibition of gluconeogenesis, increasing the number of glucose transporters and reduction of glucose absorption from the intestine [2]. One of the beneficial therapies to impair glucose absorption is through the inhibition of carbohydrate hydrolyzing enzymes such as α-amylase and α-glucosidase in the digestive organs [3,4]. Inhibitors of these enzymes delay carbohydrate digestion and prolong the overall time for carbohydrate digestion, resulting in a decrease in the rate of glucose absorption [5]. Increasing evidence has shown that prolonged exposure to elevated glucose induces the production of free radicals, particularly reactive oxygen species (ROS), through glucose auto-oxidation and protein glycosylation [6]. Oxidative injury by ROS has been suggested to explain the excess prevalence of vascular complications in diabetes mellitus, which may be mediated by oxidative stress [7,8]. An impairment in the equilibrium between ROS and antioxidants results in oxidative stress, a deleterious process that can be an important mediator of damage to cell structures, including lipids and membranes, proteins and DNA. Indeed, a variety of defects in antioxidative status have been reported in experimental and in diabetic patients when compared with normal population [9,10].

Traditional medicinal plants have been used for many years by different cultures around the world for the management of diabetes. In recent years, investigation on herbal medicines has become progressively important in the search for a new, effective and safe therapeutic agent for the treatment of diabetes. More than 200 pure bioactive principles isolated from plants have been demonstrated to have blood glucose-lowering effect [11], several of which are phenolics [12], flavonoids [13,14], triterpenoids, alkaloids [15-17] and carbohydrates [18]. *Ficus deltoidea*, a plant belonging to the Moraceae family or locally known as “Mas Cotek” is a new potential medicinal plant in Malaysia. Little is known about the chemical constituents of *F*. *deltoidea* but some of the compounds claimed to be present in *F*. *deltoidea* are the flavonoids isovitexin, vitexin [19,20], proanthocyanidins, flavan-3-ol monomers and flavones glycosides [21]. Recently, a study by Zainah and collegues proposed that the aqueous extract of *F*. *deltoidea* leaves might contain water-soluble insulin-secreting constituents with better insulin secretion activity than a well-known hypoglycaemic agent, glibenclamide [22]. Interestingly, a toxicological study on *F*. *deltoidea* reported that the plant does not contain toxic components [23]. The leaves of *F*. *deltoidea* have been shown to exhibit blood glucose-lowering effects [19,20,22,24-26], antinociceptive [27], ulcer healing [28], antioxidant [19,21,29,30], anti-inflammatory [31] and antimelanogenic [32] properties. However, to date, there is no available report on the pharmacological activities of the fruits of *F*. *deltoidea*. Therefore, the present study attempts to investigate the potential antidiabetic and antioxidant activities of two different fruit varieties from *F*. *deltoidea* (var. *angustifolia* (SF) and var. *kunstleri* (BF)) in order to develop a physiological functional food for use as antidiabetic agents.

## Methods

### Chemicals

*p*-Nitrophenyl α-D-glucopyranoside (*p*NPG), Baker’s yeast α-glucosidase, rat intestinal acetone powder, acarbose, 3,5-dinitrosalicylic acid, sucrose, *D*-(+)-glucose, 2,2-diphenyl-l-picrylhydrazyl (DPPH), 6-hydroxy-2,5,7,8-tetramethylchroman-2-carboxylic acid (Trolox), sodium carbonate, ferric chloride, ferrous sulphate, aluminium chloride, polyvinylpyrrolidone, Folin–Ciocalteau’s phenol reagent, gallic acid, quercetin, Acrylamide PAGE, 2-mercaptoethanol and ammonium sulphate were purchased from Sigma Chemical Co. (St. Louis, MO, USA). Protein assay reagent, bovine serum albumin and polypeptide standard were from Bio-Rad Laboratories (USA). Glucose oxidase kit was from Megazyme International (Ireland). All other chemicals used in this study were of analytical grade.

### Plant materials

The fruits of *F*. *deltoidea* were collected from Delto Medicama Plantation (M) Sdn Bhd., Sabak Bernam, Selangor, Malaysia. These samples included fruits from *F*. *deltoidea* var. *angustifolia* (SF) (voucher specimen: KLU 046467) and *F*. *deltoidea* var. *kunstleri* (BF) (voucher specimen: KLU 046470) were identified by the corresponding author. The fruits were cleaned, air-dried, cut into small pieces and then pulverized. Samples were deposited in the Herbarium, Rimba Ilmu, University of Malaya.

### Preparation of crude extract and fractions

The dried fruit powder of *F*. *deltoidea* (100 g) was boiled in 1 L of distilled water for 2 hours. At the end of the 2 hour boiling period, the same volume of distilled water was added and the boiling procedure was continued for another 2 hours. The crude aqueous extract was filtered, centrifuged and freeze-dried to yield the lyophilized crude aqueous extracts. Five gram of the crude aqueous extract was dissolved in 50 ml of distilled water and partitioned with 100 ml of ethyl acetate in a separating funnel. The solution was allowed to stand for 2 hours for complete solvent-water separation. The bottom aqueous layer was collected and freeze-dried, to obtain the water fraction which was free from nonpolar compounds. The ethyl acetate upper layer was then evaporated using rotary evaporator to yield the ethyl acetate fraction.

### Phytochemical screening

#### Determination of phenolic content

The phenolic content of the extracts was determined through the Folin-Ciocalteu assay developed by Singleton and Rossi [33]. Briefly, 10 μl of 1 mg/ml crude aqueous extracts or fractions were mixed with 450 μl of distilled water and 2.5 ml of 0.2 N Folin-Ciocalteu reagent. After 5 min, 2 ml of 10% sodium carbonate was added. The absorbance of the resulting blue-coloured solution was measured at 765 nm after incubation at 37°C for 30 min. Gallic acid was used as standard and phenolic content was expressed as milligram gallic acid equivalents (GAE) per gram of dried extract/fraction.

#### Determination of flavonoid content

Five hundred microlitres of 1 mg/ml crude aqueous extracts or fractions were mixed with 1.5 ml of 95% ethanol, 0.1 ml of 10% aluminum chloride hexahydrate, 0.1 ml of 1 M potassium acetate and 2.8 ml of deionized water. After incubation at room temperature for 30 min, the absorbance of the reaction mixture was measured at 415 nm. Quercetin was used as standard and the results were expressed as milligram quercetin equivalents (QE) per gram dried extract/fraction.

#### Determination of total sugar content

The sugar content in the crude aqueous extracts and water fractions were determined according to DuBois *et al*. [34], using a slightly modified phenol-sulphuric acid method. The reaction was initiated by mixing 200 μl of crude extract (or fraction) with 200 μl of 5% phenol in a test tube, followed by the addition of 2 ml of concentrated sulphuric acid. The tube was placed in hot water and kept at 90°C for 5 min. After cooling to room temperature, the absorbance of the mixture was measured at 490 nm. A series of *D*-(+)-glucose concentrations was analysed as above to develop a calibration curve. Total sugar content was calculated based on the curve, and expressed as glucose equivalents (GE) in μmol/g of dried extract or fraction.

#### Determination of protein content

Protein content of the crude aqueous extracts and water fractions were determined by Bradford protein assay with bovine serum albumin (BSA) as the standard [35]. Five microlitres of sample (or standard) was mixed with 250 μl of Bradford reagent and allowed to stand in the dark, at room temperature. After 15 min, the absorbance of each sample or standard was measured at 595 nm against a blank.

### *In vitro* antidiabetic assays

#### Yeast α-glucosidase inhibition assay

The enzymatic activities of α-glucosidase were determined colorimetrically by monitoring the release of 4-nitrophenol from 4-nitrophenyl-α-*D*-glucopyranoside (*p*NPG). Crude aqueous extracts and water fractions were dissolved in distilled water to give six concentrations while the ethyl acetate fractions were prepared using DMSO. The aqueous extracts and fractions (125 μl) were pre-incubated with 250 μl of 0.1 U/ml α-glucosidase at 37°C for 5 min. On the other hand, 25 μl of the ethyl acetate fraction was mixed with 100 of distilled water before pre-incubation with the enzyme solution. The reaction was initiated by adding 125 μl of freshly prepared *p*NPG to the mixture followed by 30 min incubation at 37°C. Reaction was terminated by the addition of 1 ml of 0.1 M Na_2_CO_3_. Assays using acarbose as the positive control were also carried out as above. Absorbance values were recorded at 405 nm and the α-glucosidase inhibitory activity (%) was calculated as follows:

$$\%\;\mathrm{inhibition}=\frac{{\mathrm{Abs}}_{\mathrm{control}}-{\mathrm{Abs}}_{\mathrm{extract}}}{{\mathrm{Abs}}_{\mathrm{control}}}\times 100$$

The IC_50_ value, defined as the concentration of the samples causing 50% inhibition of α-glucosidase was estimated by non-linear regression analysis using Graph Pad Prism software.

#### Rat intestinal α-glucosidase inhibition assay

The inhibitory activity of *F*. *deltoidea* against α-glucosidase was determined using sucrose and 4 mM *p*NPG, and as the substrates. The reaction mixture consisted of 50 μl of the extract or fraction, 25 μl of enzyme solution and 50 μl of sodium phosphate buffer in a tube. Acarbose was used as the positive control. After 5 min of pre-incubation at 37°C, reaction was initiated by adding 50 μl of 50 mM sucrose or 4 mM *p*NPG. The mixture was further incubated at 37°C for 45 min (sucrose) or 30 min (*p*NPG). Reaction was terminated by adding 0.25 ml of 0.1 M of Na_2_CO_3_ and the release of *p*-nitrophenol from *p*NPG was measured at 405 nm. When sucrose was used as the substrate, reaction was stopped by incubating each tube in boiling water bath for 5 minutes. The concentrations of glucose released from sucrose were determined by mixing 250 μl of the solution with glucose oxidase reagent. After 20 min of incubation, absorbance was read at 415 nm. Inhibition rates were calculated based on the previous equation and the IC_50_ values were determined. All experiments were carried out in triplicates and the results are expressed as the mean ± S.D. of three determinations.

#### α-amylase inhibition assay

The crude aqueous extracts and fractions (125 μl) with different concentrations were incubated with 125 μl of α-amylase solution (1 U/ml phosphate buffer) at 37°C for 10 min. After pre-incubation, 125 μl of 0.5% starch solution was added into the tube and further incubated for 30 min. The reaction was stopped by the addition of 250 μl of dinitrosalicylic acid reagent and immediately tubes were incubated for 5 min in a boiling water bath. Once cooled to room temperature, the mixture was diluted with 1.5 ml of distilled water and the absorbance was measured at 540 nm. Acarbose was used in the assay as a positive control. The percentage of α-amylase inhibition and IC_50_ value were calculated using the same equation as for α-glucosidase inhibition.

### *In vitro* antioxidant assays

#### 2, 2-diphenyl-1-picrylhydrazyl (DPPH) radical scavenging activity

The scavenging activity of the stable free radical, DPPH• was determined according to the method described by Braca *et al*. [36]. DPPH solution was prepared by diluting 20 μl of DPPH stock solution (2 mg/ml methanol) with 0.98 ml of methanol. Samples were added to the diluted DPPH solution in a 1:6, volume to volume ratio. A series of concentrations ranging from 25 to 1000 μg/ml samples were tested. The mixtures were shaken vigorously and incubated in the dark for 30 min after which the reduction of DPPH• absorption was measured at 517 nm. The percentage of inhibition was calculated according to the following equation:

$$\%\;\mathrm{inhibition}=\frac{{\mathrm{Abs}}_{\mathrm{control}}-{\mathrm{Abs}}_{\mathrm{extract}}}{{\mathrm{Abs}}_{\mathrm{control}}}\times 100$$

Quercetin was used as a positive control. Scavenging activity of the plant extracts was estimated based on the percentage of the DPPH• reduction by calculating the IC_50_ values of DPPH radicals using a non-linear regression analysis.

#### Ferric reducing ability of plasma (FRAP) assay

The ferric reducing activity of the plant samples was estimated based on the ferric reducing ability of plasma (FRAP) procedure described by Benzie and Strain [37]. Working FRAP reagent was prepared by mixing 25 ml acetate buffer, 2.5 ml 2, 4, 6-tripyridyl-s-triazine (TPTZ) solution and 2.5 ml of FeCl_3_.6H_2_O solution. The assay was performed by incubating the freshly prepared FRAP reagent at 37°C for 5 min and the blank reading was taken at 593 nm. Thereafter, crude aqueous extracts, fractions, or standard along with distilled water was added to the FRAP reagent at a ratio of 1:3:30 (volume to volume), respectively. Absorbance was recorded at 0 sec immediately upon addition of the FRAP reagent and every 15 sec for 4 min. The assay was repeated using varying concentrations of FeSO_4_.7H_2_O to construct the standard curve. Calculated FRAP values were expressed as mmol ferric ions reduced by the samples to the ferrous form, per gram of dried extract/fraction. Quercetin was used as positive control.

#### Superoxide anion radical scavenging activity

Superoxide anion radical scavenging activity was performed according to the method of Nikishimi and colleagues [38]. Superoxide radicals were determined by the phenazine methosulphate (PMS)-NADH superoxide generating system. The reaction mixture contained 25 μl of sample, 50 μl of nitroblue tetrazolium (NBT), 50 μl of NADH and 50 μl of 120 μM PMS. NBT, NADH and PMS solutions were prepared in 0.1 M sodium phosphate buffer (pH 7.4). The mixture was incubated at room temperature for 30 min and the absorbance read at 560 nm. The decrease of absorbance at 560 nm was calculated as percentage of inhibition of NBT using the same equation as the DPPH assay and expressed as mg of Trolox equivalent (TE) per gram of dried aqueous extract/fraction. Quercetin was used as positive control.

#### 2, 2′-azinobis (3-ethylbenzothiazolinesulphonic acid) (ABTS) radical scavenging activity

The ABTS radical cation (ABTS^•+^) was generated by mixing equal volume of ABTS solution and potassium persulfate, and allowing them to react for 12–16 h in the dark at room temperature. The solution was diluted with methanol to give an absorbance of 0.70 (± 0.02) at 734 nm prior to use. Three microlitres of sample was reacted with 300 μl of fresh ABTS^•+^ solution and absorbance was measured at 734 nm after 15 min incubation in the dark. Trolox standard curve was generated and the ABTS radical scavenging capacity was expressed as mmol Trolox equivalent (TE) per gram of dried aqueous extract/fraction. Quercetin was used as positive control.

#### Nitric oxide radical scavenging activity

Fifty microlitres of sample was mixed with 200 μl of SNP in PBS and was incubated in the dark at room temperature. After 150 min, 25 μl of Griess reagent was added to the reaction mixture and further incubated for 30 min. The pink chromophore generated during diazotization of nitrite ions with sulphanilamide and subsequent coupling with NED was measured spectrophotometrically at 540 nm against a blank sample. Results were expressed as percentage inhibition of the nitric oxide radicals. The nitric oxide radical scavenging activity of the extracts was also measured using the Trolox standard curve and results were expressed as mmole Trolox equivalent (TE) antioxidant capacity per gram of dried extract/fraction.

##### Sodium dodecyl sulfate-polyacrylamide gel electrophoresis (SDS-PAGE) analyses

The crude aqueous extracts and water fractions were treated with 1% insoluble polyvinylpyrrolidone (PVP) and stirred continuously at 4°C for 4 hours. PVP-bound phenolics were removed by centrifugation at 10 000 rpm and the supernatant was next precipitated with ammonium sulfate (90% saturation) at room temperature. Following centrifugation at 10 000 rpm, the precipitated proteins were dissolved in 5 ml distilled water and dialyzed against distilled water (4°C, 48 h, three changes). Protein precipitation was furthered using cold acetone according to the method of Sindhu *et al*. [39]. The final protein content in the sample solutions was quantified and then lyophilized before storage at −20°C. Proteins were analyzed by SDS-PAGE according to the method of Laemmli [40]. The crude extracts and water fractions containing 5 μg protein, along with a peptide standard (MW 1.4-27 kDa) were heated at 95°C for 4 minutes in SDS-PAGE sample buffer containing 5% β-mercaptoethanol. Fifteen microlitre of samples and five microlitre of the marker were loaded into the sample wells of 4% stacking gel. Electrophoresis was performed using 18% Tris-tricine gel at an initial voltage of 60 V for 10 min and then increased to a constant voltage at 150 V, once samples had entered the separating gel. The bands were visualized by silver staining and the relative mobility (R_f_) of each band in the standards and the samples was calculated using the following equation:

$$R_{f}=\frac{\mathrm{Distance}\;\left(\mathrm{in}\;\mathrm{mm}\right)\;\mathrm{protein}\;\mathrm{migrated}}{\mathrm{Distance}\;\left(\mathrm{in}\;\mathrm{mm}\right)\;\mathrm{of}\;\mathrm{Coomassie}\;\mathrm{blue}\;\mathrm{dye}\;\mathrm{front}}$$

Using the molecular weight of protein standards, log MW (y-axis) versus R_f_ (x-axis) was plotted to generate a calibration curve against which the molecular weight of unknown protein samples were determined.

##### Surface enhanced laser desorption/ionization time-of-flight mass spectrometry (SELDI-TOF MS)

All ProteinChip arrays were pre-treated according to manufacturer instructions. The reversed-phase hydrophobic surface ProteinChip array (H50) were applied with 2.5 μl binding buffer (0.5% trifluoroacetic acid/50% acetonitrile) and allowed to air dry. Four microlitre of sample (1 mg/ml), was applied to each spot and the array was incubated at room temperature for 30 min. Next, 2 μl of concentrated matrix solution (7 mg α-cyano-4-hydroxycinnamic acid/ml binding buffer) was added to each spot. The ProteinChip array was air-dried (30 min) and analysed in a ProteinChip Reader (BioRad Laboratories, USA). Each sample was bound to each array surface in triplicate. The SELDI analysis was performed according to the protocol of Lau and colleagues [41].

### Statistical analysis

All experimental results were expressed as means of triplicate analysis ± standard deviations. Differences between groups were analysed using Student’s *t*-test on theMicrosoft Excel software. Correlation analyses were calculated using Pearson correlation analysis SPSS software, version 17 (SPSS Inc., Chicago, IL). A value of *p* < 0.05 was considered statistically significant.

## Results

### Phenolic and flavonoid content

Phenolic compounds are secondary metabolites ubiquitously found in plants, mainly acting as UV protectors. For both varieties, the crude extracts (C) showed the highest phenolic content compared to the fractions (Table 1). Following fractionation, the water fractions (W) from both varieties showed higher phenolic content than the ethyl acetate fractions (E). Flavonoid content was higher in the crude extract of var. *angustifolia* (SFC) and its fractionation led to higher flavonoids in the ethyl acetate fraction (SFE) compared to the water fraction (SFW). In contrast, the ethyl acetate fraction of var. *kunstleri* (BFE) had higher flavonoid content than the corresponding crude extract. Statistical analyses showed that there were significant differences in phenolic content between extracts and the fractions of the same variety (*p* < 0.01).

**Table 1:** **Phytochemical constituents of the crude aqueous extracts and fractions of the different fruit varieties of *F. deltoidea***

***F. deltoidea***	**Total flavonoid content (mg quercetin equivalents/g dried extract/fraction)**	**Total phenolic content (mg gallic acid equivalents/g dried extract/fraction)**	**Sugar (μmol glucose/g dried extract/fraction)**	**Protein (μg protein/mg dried extract/fraction)**
**SF** (var. *angustifolia*)				
**SFC**	17.34 ± 0.63^*a*^	121.62 ± 4.86^*f*^	2.58 ± 0.03^*l*^	46.67 ± 1.67^*p*^
**SFE**	16.51 ± 0.60^*a*^	47.09 ± 1.68^*g*^	ND	ND
**SFW**	12.37 ± 1.15^*b*^	59.29 ± 1.51^*h*^	1.83 ± 0.05^*m*^	56.11 ± 5.36^*p*^
**BF** (var. *kunstleri*)				
**BFC**	26.65 ± 1.00^*c*^	96.89 ± 2.57^*i*^	2.35 ± 0.03^*n*^	57.78 ± 3.85^*q*^
**BFE**	12.31 ± 2.19^*d*^	36.27 ± 2.98^*j*^	ND	ND
**BFW**	7.46 ± 0.54^*e*^	90.62 ± 1.27^*k*^	2.45 ± 0.05^*n*^	73.33 ± 4.99^*r*^

Each value is expressed as a mean ± SD (n = 3). Values with different superscript letters between the same variety, within the same column are significantly different at *p* < 0.01. ND: not determined. SFC, SFE and SFW indicate crude aqueous extract, ethyl acetate fraction and water fraction of SF (var. *angustifolia*). BFC, BFE and BFW indicate crude aqueous extract, ethyl acetate fraction and water fraction of BF (var. *kuntsleri*).

### Estimation of sugar and protein contents

As shown in Table 1, both SFC and BFC contained almost similar concentrations of sugar. Fractionation slightly reduced the sugar content of SFW while there was no significant difference in the sugar content between BFC and BFW. Apart from that, SFW and BFW contained more proteins than the corresponding crude extracts (Table 1) with only BF showing significant difference (*p* < 0.01).

### Inhibition of α-glucosidase activity

The α-glucosidase inhibitor potencies of extracts and fractions of the fruit varieties were compared on the basis of their IC_50_ values (Table 2). The BFW was the most potent inhibitor of yeast α-glucosidase activity (48.50 ± 3.43 μg/ml) when *p*-nitrophenyl-α-D-glucopyranoside (*p*NPG) was used as the substrate. BFC and its corresponding ethyl acetate fraction suppressed the activities of α-glucosidase better than the SF samples. Acarbose, the positive control used in this study weakly inhibited yeast α-glucosidase with IC_50_ value of 9.075 ± 0.065 mg/ml. When crude mammalian α-glucosidase was used, with sucrose as the substrate, the IC_50_ values were significantly increased. BFW again inhibited α-glucosidase the most with IC_50_ value of 444.43 ± 3.01 μg/ml. When *p*NPG was used as the substrate for rat intestinal α-glucosidase, the IC_50_ values further increased, suggesting its selective inhibition towards different substrate. The IC_50_ values of the positive control acarbose towards glycosidic bond cleavage of sucrose and *p*NPG by rat intestinal α-glucosidase were 39.45 ± 0.499 and 57.79 ± 0.66 μg/ml respectively.

**Table 2:** **Inhibition of yeast and mammalian α-glucosidases by the crude aqueous extracts and fractions of the fruits of *F. deltoidea *from two different varieties**

**Substrate**	**α-glucosidases inhibitory activities, IC**_**50 **_**(mg/ml)**						
	**SF (var. *angustifolia*)**			**BF (var. *kunstleri***)			**Acarbose**
	**SFC**	**SFE**	**SFW**	**BFC**	**BFE**	**BFW**	
Yeast α-glucosidase							
*p*NPG	0.065 ± 0.005^*a*^	0.259 ± 0.014^*b*^	0.083 ± 0.006^*a*^	0.061 ± 0.003^*c*^	0.225 ± 0.008^*d*^	0.049 ± 0.003^*e*^	9.075 ± 0.065
Rat intestinal α-glucosidase							
Sucrose	0.552 ± 0.013^*a*^	0.611 ± 0.020^*a*^	0.473 ± 0.011^*b*^	0.538 ± 0.016^*c*^	0.596 ± 0.015^*c*^	0.444 ± 0.004^*d*^	0.039 ± 0.001
*p*NPG	7.226 ± 0.389^*a*^	13.957 ± 0.238^*b*^	7.173 ± 0.170^*a*^	6.407 ± 0.102^*c*^	10.040 ± 0.291^*d*^	4.123 ± 0.136^*e*^	0.058 ± 0.001

Each value is expressed as a mean ± SD of triplicates from three different experiments. Values with different superscript letters between the same variety, within the same row are significantly different at *p* < 0.01. SFC, SFE and SFW indicate crude aqueous extract, ethyl acetate fraction and water fraction of SF (var. *angustifolia*). BFC, BFE and BFW indicate crude aqueous extract, ethyl acetate fraction and water fraction of BF (var. *kuntsleri*).

### α-amylase inhibitory activity

Repeated α-amylase assays however demonstrated that none of the extracts or the fractions exhibited inhibitory activity towards α-amylase under the test condition. Acarbose however, strongly suppressed the activity of α-amylase, giving an IC_50_ of 7.02 ± 0.08 μg/ml.

### Antioxidant activities

Comparing the two varieties, SFC has better antioxidant activities than the BFC although their activities were lower than the positive control quercetin (Table 3). In general, both the crude extracts revealed greater antioxidant capabilities than the fractions. Following fractionation, antioxidant activities in SF and BF were found to be distributed between the two fractions.

**Table 3:** **Antioxidant activities of the crude aqueous extracts and fractions of the fruits of *F. deltoidea***

**Antioxidant assays**	**SF (var. *angustifolia*)**			**BF (var. *kunstleri*)**			**Quercetin**
	**SFC**	**SFE**	**SFW**	**BFC**	**BFE**	**BFW**	
DPPH radical scavenging activity, IC_50_ (μg/ml)	111.20 ± 4.77^*a*^	678.18 ± 22.31^*b*^	195.75 ± 12.19^**c**^	150.25 ± 3.05^*d*^	499.93 ± 5.10^*e*^	148.02 ± 2.25^*d*^	11.76 ± 0.57
Superoxide radical scavenging activity, IC_50_ (μg/ml)	141.33 ± 1.70^*a*^	145.15 ± 2.92^*a*^	453.13 ± 3.76^*b*^	153.92 ± 0.76^*c*^	212.50 ±3.01^*d*^	163.78 ± 1.98^*e*^	60.52 ± 2.82
NO radical scavenging activity, IC_50_ (mg/ml)	0.79 ± 0.01^*a*^	1.47 ± 0.06^*b*^	1.68 ± 0.02^*c*^	1.24 ± 0.03^*d*^	2.02 ± 0.06^*e*^	1.58 ± 0.02^*f*^	101.02 ± 1.18
Ferric reducing capacity	1.82 ± 0.19^*a*^	0.34 ± 0.11^*b*^	0.62 ± 0.06^*c*^	1.27 ± 0.11^*d*^	0.23 ± 0.08^*e*^	0.77 ± 0.08^*f*^	5.70 ± 0.05
(mmole of Fe^2+^/g of dried extract/fraction)							
ABTS radical scavenging activity (mmole Trolox/g of dried extract/fraction)	1.01 ± 0.04^*a*^	0.39 ± 0.01^*b*^	0.36 ± 0.05^*b*^	0.88 ± 0.07^*c*^	0.38 ± 0.01^*d*^	0.82 ± 0.08^*c*^	18.74 ± 0.89

Each value is expressed as a mean ± SD of triplicates from three different experiments. Values with different superscript letters between the same variety, within the same row are significantly different at *p* < 0.01. SFC, SFE and SFW indicate crude aqueous extract, ethyl acetate fraction and water fraction of SF (var. *angustifolia*). BFC, BFE and BFW indicate crude aqueous extract, ethyl acetate fraction and water fraction of BF (var. *kuntsleri*).

### Correlation analyses

Statistical analysis showed no correlation between phenolic and flavonoid content of the extracts and fractions (r^2^ < 0.0216). No correlation was also observed between the α-glucosidase inhibitory activities and both the phenolic (r^2^ < 0.5837) and flavonoid (r^2^ < 0.5459) content in the extracts and fractions. A significant positive correlation was seen between radical scavenging activities and phenolic content of the crude extracts but there was no correlation between antioxidant activities and the amount of flavonoids in the extracts or fractions.

#### Effect of protein concentration and phenolic removal on the antidiabetic and antioxidant activities of the fruits of *F*. *deltoidea*

The protein in the fruits was concentrated using ammonium sulphate and cold acetone precipitation whereas phenolics were removed with PVP. Following this treatment, the protein content of the crude extracts and water fractions increased compared to the nontreated samples (Table 4). In contrast, the phenolic content significantly decreased in samples treated with PVP. Repeated rat intestinal α-glucosidase inhihibitory activity using sucrose as the substrate showed that the IC_50_ values of the fruit samples decreased significantly (*p* < 0.05) compared to the nontreated samples (Table 4). On the other hand, DPPH radical scavenging assays on the treated samples resulted in significantly higher IC_50_ values (*p* < 0.01), indicating lower scavenging activities compared to the nontreated fruit samples.

**Table 4:** Effects of protein concentration and phenolic removal on α-glucosidase and DPPH radical scavenging activities

***F. deltoidea *samples**	**Total phenolic content (mg gallic acid equivalent/g sample)**	**Protein (μg protein/mg sample)**	**IC**_**50**_**, (μg/ml)**	
			**Crude α-glucosidase inhibitory activity**	**DPPH radical scavenging activity**
SF (var. *angustifolia*)				
SFC	5.89 ± 0.12^a,***^	49.53 ± 3.06^*e*^	348.38 ± 20.06^**^	547.83 ± 18.11^***^
SFW	3.98 ± 0.07^b,***^	59.53 ± 1.16^*e*^	396.99 ± 16.01^*^	975.20 ± 20.79^***^
BF (var. *kunstleri*)				
BFC	8.29 ± 0.10^c,***^	70.87 ± 1.15^f,*^	350.72 ± 16.80^**^	449.64 ± 17.3^**^
BFW	6.56 ± 0.12^d,***^	86.86 ± 3.10^g,**^	199.64 ± 10.77^***^	531.17 ± 21.59^***^

Each value is expressed as a mean ± SD (n = 3). Values with different superscript letters between the same variety, within the same column are significantly different at *p* < 0.01. *p < 0.05, **p < 0.01 and ***p < 0.001 are significant differences from samples which were not treated with PVP, (NH_4_)_2_SO_4_ and cold acetone. PVP: Polyvinylpyrrolidone; (NH_4_)_2_SO_4_: ammonium sulphate. SFC and SFW indicate crude aqueous extract and water fraction of SF (var. *angustifolia*). BFC and BFW indicate crude aqueous extract and water fraction of BF (var. *kuntsleri*).

#### SDS-PAGE analyses of the fruits of *F*. *deltoidea*

**Figure 1:**
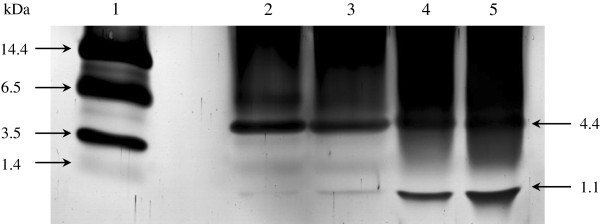
**SDS-PAGE analysis of proteins in *F. deltoidea *fruit samples.** Lane 1: peptide markers, lane 2: SFC - crude aqueous extract (var. *angustifolia*), lane 3: SFW - water fraction (var. *angustifolia*), lane 4: BFC - crude aqueous extract (var. *kunstleri*), lane 5: BFW - water fraction (var. *kunstleri*).

As shown in Figure 1, the SDS-PAGE profile of *F*. *deltoidea* protein fractions exhibited a clear peptide band appearing at approximately 4.4 kDa. The bands of SFC and SFW however were more distinct than those from BF samples. Each band however, might not necessarily correspond to a single protein because a few proteins with almost similar molecular weights may exist in one band.

### Protein detection by SELDI-TOF MS

**Figure 2:**
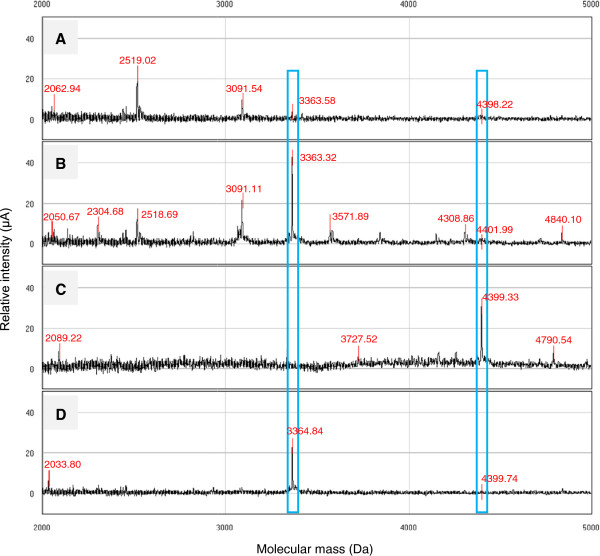
**SELDI-TOF MS spectra showing the protein profiles of *F. deltoidea *fruit samples. A**: BFC - crude aqueous extract (var. *kunstleri*), **B**: BFW - water fraction (var. *kunstleri*), **C**: SFC - crude aqueous extract (var. *angustifolia*), **D**: SFW - water fraction (var. *angustifolia*). Square boxes indicate the common protein peaks found in the crude aqueous extracts and water fractions of both varieties.

The SELDI-TOF MS spectra generated from the crude extracts of both varieties demonstrated different protein profiles (Figure 2A and C). BFC showed the presence of several protein peaks with *m*/*z* values lower than 3360 whereas SFC exhibited the opposite trend. Protein peaks corresponding to *m*/*z* values of 3360 and 4400 were observed in both samples, however, with low relative intensities. The peak at 4400 Da coincided well with the band on SDS-PAGE but its relative intensity value did not correlate with the intensity of the band. The protein patterns of BFC displayed changes in the protein peak intensities following fractionation. Peak intensities at *m*/*z* values of ~3090, 3360 and 4400 were shown to be significantly increased. Several other peaks which were present in low abundance at *m*/*z* ~2300, 3500, 3800, 4100, 4300 and 4800 became more apparent in BFW (Figure 2A and B). On the other hand, SF showed different protein profile whereby most of the peaks observed in SFC were absent following fractionation, with exception for a protein with *m*/*z* 3360.

## Discussion

The antidiabetic and antioxidant activities of *F*. *deltoidea* have been reported in various studies but the part used in most of the studies was the leaf. This study was the first attempt to evaluate the ability of the fruit aqueous extracts and fractions form different varieties of *F*. *deltoidea* to act as antidiabetic and antioxidant agents. Partitioning of the crude aqueous extracts with ethyl acetate was aimed at separating the non-polar secondary metabolites from the highly polar compounds in the water fraction. Moreover, fractionation techniques also serves as a method of concentration of the polar components as observed in the increase of protein content in the water fraction. In general, the results of the phenolic and flavonoid contents indicated that these compounds were present at lower amounts in the aqueous extracts but could be explained by the fact that these extracts were obtained at high temperature of extraction.

Studies have reported positive correlation between phenolic content of plants and their respective antidiabetic activities [42,43]. In contrast to the work of Farsi *et al*., [19], the correlation analyses showed that phenolics and flavonoids were not directly involved in the α-glucosidase inhibitory activities. The water fractions of both varieties of *F*. *deltoidea*, as a whole were better inhibitors of α-glucosidase compared to the ethyl acetate fractions and the crude aqueous extracts. The use of acarbose as the inhibitor of α-glucosidase and α-amylase did not show any sign of inactivation within the range of concentration tested. This is in good agreement with other studies which stated little inhibition [44,45], or no inhibitory activity of acarbose [46-48] towards yeast α-glucosidase. Many of the considerably potent inhibitors of baker’s yeast α-glucosidase possessed lower inhibitory activities against mammalian α-glucosidase and is suggested to be due to the difference of molecular recognition in the binding site of the enzyme [49,50]. Negative inhibition values obtained for all extracts against porcine pancreatic amylase indicated that the enzyme activity was accelerated rather than suppressed. Thus, this suggests that the glucose-lowering property of *F*. *deltoidea* is achieved through inactivation of α-glucosidase but not via α-amylase inhibition.

Studies have shown that antioxidants play a key role in reducing diabetic complications [51,52]. Furthermore, positive correlation between α-glucosidase inhibition and antioxidant activity has been reported in numerous studies [45,53]. Various antioxidant assays have been developed for the estimation of antioxidative properties of plants. Ideally, a combination of assays, incorporating different mechanisms of action is useful in order to provide complete information on the antioxidant capacity of a particular plant. Fractionation of the aqueous extracts with ethyl acetate was shown to reduce the antioxidant activities. This proposes that the bioactive principles in the crude extracts may act synergistically to produce the antioxidant effect, and fractionation might have removed some of the compounds. Indeed, synergistic effects of several antioxidants in plants have been previously reported [54,55].

In order to further evaluate the effect of protein and phenolics in the fruits on antidiabetic and antioxidant activities, protein in the fruits were concentrated using ammonium sulphate and cold acetone whereas phenolics were removed using a polymer, polyvinylpyrrolidone (PVP). Ammonium sulphate precipitation is the most commonly used salt precipitation because the amount of precipitated protein will be large enough to be visible on the SDS PAGE gel. Furthermore, this technique stabilizes most proteins in the solution and reduces the lipid content resulting in a better protein profile on the gel. In the present study, the concentrated proteins were obtained in 90% saturated ammonium sulfate precipitate. On the other hand, binding of PVP to the hydroxyl group in the phenolic compounds through hydrogen bonding removes phenolics from the extracts and water fractions. The effective removal of phenolic compounds however depends on the number of hydroxyl group present that will affect the degree of PVP binding to phenolics [56]. The results indicating increased protein content as well as decreased activities of α-glucosidase activities shown by the treated *F*. *deltoidea* samples strongly justified the important role of proteins in exhibiting the antidiabetic property. As statistical analyses failed to correlate phenolics and α-glucosidase inhibitory activities, other highly polar phytochemicals may have contributed to these effects. Plant components other than phenolics, such as polysaccharides [57,58] or peptides [59,60] have been shown to possess antidiabetic activities and these may have contributed to the observed inhibition. It is also evident from the literature that some plants contain insulin-like peptides which give the hypoglycemic ability [61]. Apart from that, significant decreases in the phenolic contents and DPPH radical scavenging activities of the treated samples implies the major contribution of phenolics to the total antioxidant capacity. Correlation analyses between phenolic content and radical scavenging activities of the crude extracts, together with findings reporting positive correlation between plant phenolics and antioxidant activity [62,63], further support this observation. Although plant flavonoids have been indicated to positively correlate with antioxidant activities [64], our study found no correlation between the antioxidant activities and the amount of flavonoids in the extracts or fractions.

SDS-PAGE analysis of the proteins was carried out using Tris-tricine gel due to the low molecular weight proteins presence in the fruit samples. In addition, proteins with molecular mass lower than 20 kDa is well suited to be profiled by SELDI-TOF MS [65]. In our study, a surface-enhanced laser desorption/ionization time of flight (SELDI-TOF)-based ProteinChip System was used for rapid identification of protein patterns in SF and BF. The reversed-phase H50 ProteinChip® array used in this study binds proteins by hydrophobic interaction, thus hydrophilic proteins will not bind properly to the arrays which may lead to poor detection. The binding of proteins to the surface of the array can also be influenced by the amount of ACN in the binding buffer where higher ACN restricts the binding to proteins which are more hydrophobic than the buffer [65]. SELDI-TOF MS spectra showed the presence of several low molecular mass proteins in both crude extracts and their respective water fractions. The spectra is somewhat different between the two Ficus varieties, hence, could suggest the suitability of SELDI-TOF MS profiling of proteins as a new identification tool to distinguish SF from BF. Further analysis on the SELDI-TOF MS profile demonstrated that two protein peaks with the *m*/*z* values ~3360 and 4400 are commonly found in both varieties. This finding could also serve as potential identification marker for the screening and finger-printing of *F*. *deltoidea* plant. In view of the antidiabetic evaluation; both water fractions demonstrated significant activity and both contained protein peaks 3360 and 4400 Da at varying intensities. These further suggest that those low molecular mass proteins could be involved in the observed antidiabetic effect. However, the extent of their role and function requires further characterization step as well as protein identification.

## Conclusion

The results obtained are in support of folklore uses of *F*. *deltoidea* in reducing blood sugar levels. Although the leaves are the parts that are traditionally used, in this study we demonstrated that both the fruit varieties also showed effective antidiabetic properties. Overall, BFW and SFW had greater antidiabetic effects compared to the corresponding crude aqueous extracts or ethyl acetate fractions, implying that compounds responsible for these activities are polar in nature. SFC was a better antioxidant than BFC and this was probably due to the phenolic compounds. Proteomics analysis by means of SDS-PAGE and SELDI-ToF demonstrated the presence of several low molecular mass proteins in the range of 3360 and 4800 Da in the active water fractions. The involvement of carbohydrates and proteins in the fruits with the observed biological effects however, remains unclear. Animal studies are currently on going to investigate if the *in vitro* observations could be replicated *in vivo*. This would provide further information on the potential use of the fruits of *F*. *deltoidea* as an antidiabetic agent and their relationship with its antioxidant activity.

## Competing interest

The authors declare that they have no competing interests.

## Authors’ contributions

HM performed all experimentations analysed the data and wrote the manuscript. AAA designed the antioxidant study and supervised the experimental work. NHA designed the antidiabetic, protein and proteomics studies and supervised the experimental work. All authors read and approved the final manuscript.

## Pre-publication history

The pre-publication history for this paper can be accessed here:

http://www.biomedcentral.com/1472-6882/13/118/prepub
